# Engaging for change: the influence of civic engagement on at-risk Arab minority youth in Israel

**DOI:** 10.3389/fpsyg.2026.1711399

**Published:** 2026-04-20

**Authors:** Serene Dakak-Abed Al Wahad, Lana J. Jeries Loulou, Limor Goldner, Galit Yanay Ventura

**Affiliations:** 1 School of Creative Arts, Emily Sagol Cats Research Center, University of Haifa, Haifa, Israel; 2 Paul Baerwald School of Social Work and Social Welfare, Hebrew University of Jerusalem, Jerusalem, Israel; 3 Department of Human Resources, Max Stern Yezreel Valley College, Jezreel Valley, Israel

**Keywords:** Arab citizens of Israel, civic engagement, minority groups, social involvement, young adults

## Abstract

**Aims:**

This study examined how an organized civic engagement program affected socially excluded young Arab adults in Israel, who face systemic discrimination, political alienation, and socioeconomic disparities.

**Method:**

The sample included 132 participants (*M*_age_ = 20.40, *SD* = 2.04, 57% women) from the Meidad Enterprise-Social Engagement project. Using a longitudinal pre–post design with repeated MANOVA analyses, we assessed changes in civic attitudes, behaviors, and wellbeing. Additionally, K-means cluster analyses, with repeated-measures MANOVAs, measured civic engagement gains among subgroups based on baseline wellbeing, depression, and loneliness.

**Results:**

The results indicated significant gains in civic engagement attitudes, perceptions, and skills (*μ*^2^ = 0.13), civic engagement behaviors (*μ*^2^ = 0.09), and wellbeing (*μ*^2^ = 0.08). Participants with higher self-reported depressive symptoms and loneliness, in addition to lower levels of self-worth, life satisfaction, meaning, and hope, demonstrated the most pronounced improvements over time.

**Conclusion:**

Findings suggest organized civic engagement could benefit marginalized minority youth, especially those with moderate psychopathology and low wellbeing.

## Introduction

Emerging adulthood, which is typically defined as the period between 18 and 30, is characterized by the exploration of identity and life choices (Wood et al., 2017). During this time, individuals often question the beliefs, norms, and values formed during their adolescence, which can shape their future trajectories (Gutiérrez-García et al., 2018). By the end of this period, individuals are expected to make critical decisions about education, career paths, and other significant areas of life (Arnett, 2000, 2007). Research indicates that addressing developmental challenges such as career preparation through higher education and cultivating friendships, predicts long-term success in professional and interpersonal domains (Roisman et al., 2004). However, contemporary challenges such as economic instability and complex identity formation often delay full independence until the late twenties or early thirties (Arnett, 2002) and are accompanied by emotional distress, including identity crises, future-related anxieties, pessimism, and feelings of isolation, as well as increased engagement in risky behaviors (Côté, 2014; Sulimani-Aidan, 2020; Schwartz, 2016).

Critics contend that definitions of emerging adulthood predominantly mirror the experiences of the White middle class in Western developed nations (Bynner, 2005; Hendry and Kloep, 2007) and fail to comprehensively capture the experiences of minority groups (Syed et al., 2011, 2013). Furthermore, in many industrialized nations, race and social class intersect to produce unequal access to educational, economic, and civic resources, which exposes racial and ethnic minority emerging adults to structural barriers that constrain normative pathways of exploration (Bialik and Fry, 2019; Côté, 2006, 2014; Syed et al., 2013). As a result, minority youth often assume adult roles at younger ages. Limited resources increase the risk of long-term marginalization that has enduring consequences for life satisfaction and wellbeing (McLean et al., 2016; Ristikari et al., 2018; Greene and Patton, 2020). Despite these structural barriers and adverse experiences, racial and ethnic minority youth often sustain strong aspirations for higher education and identity development, thus underscoring both their resilience and the need for supportive developmental contexts (Arnett and Schwab, 2012; Arnett and Tanner, 2011).

### Emerging adulthood of young adult Arabs living in Israel

The Indigenous Arab minority in Israel (also known as the Palestinian-Arab community in Israel or Israeli Arabs), which constituted the majority of the population in Palestine until the establishment of the State of Israel in 1948 (Kimmerling, 2004), faced restrictions on their human rights under martial law until 1966, since they were viewed as the enemy by the Israeli government. Today this minority must still cope with extreme socio-political challenges, including minority stress, racism, microaggressions, and exclusion. These layered forms of exclusion and discrimination are further exacerbated by discriminatory policies, such as the nation-state law passed by the Knesset in 2018, which designates Israel as the nation-state of the Jewish people alone. It grants collective national rights exclusively to Jews, and downgrades Arabic as a national language, thus reinforcing the already considerable structural inequalities faced by Arab citizens. This legislation exacerbates political alienation and discrimination across crucial sectors, including employment, income, housing, health, and welfare (Ben-Porat et al., 2021; Jabareen, 2018; Alarcon et al., 2013), constrains civic engagement opportunities, and contributes to lower wellbeing, all of which point to the need for targeted interventions to empower Arab youth in Israel.

According to the Israel Central Bureau of Statistics (Granit, 2021), young Arabs living in Israel make up 22% of the 18–25 age bracket. Indigenous Arabs represent the three major religious groups of Muslims, Christians, and Druze. These communities face economic and social marginalization characterized by higher poverty rates, fewer resources, and lower labor-market integration than Israeli society as a whole (Chaider et al., 2010; Miaari and Hadad Haj-Yahya, 2017). Although young Arab adults in Israel share the uncertainties of emerging adulthood with their peers, they also face racism, socioeconomic disparities, discrimination in education, and marginalization (Mahajna, 2017; Tanner, 2015).

Compared to their Jewish counterparts, Arab youth in Israel encounter significant structural barriers that restrict civic participation in numerous facets of life. Arab youth have notably higher secondary school dropout rates (24%) than Jewish youth (7–8%) and enroll much less in higher education. Only approximately 20–25% register or complete their tertiary education, as compared to 48–52% of Jewish young adults. Economic marginalization is also more pronounced among Arab youth, with approximately 43% of their families living below the poverty threshold, compared to about 13–15% in Jewish youth. Arab youth also have lower employment security (30% versus 14%), experience higher levels of psychological distress, and report significantly lower life satisfaction, with roughly 22% expressing dissatisfaction with life compared to 4% among Jewish youth (Kahn-Strawczynski et al., 2016; Mahajna, 2017; Sulimani-Aidan, 2020, 2022).

Studies attribute these challenges to structural inequalities, institutional discrimination, and the quasi-total absence of integration into Jewish societal frameworks. These disparities manifest in unequal access to education and the lack of structured transitional pathways, such as military service or gap-year programs that typically provide Jewish youth with direction and social integration (Jeries-Loulou and Khoury-Kassabri, 2022; Mahajna, 2017). Other factors, including the leadership crises within Arab society, community violence, and the ongoing effects of political conflict, further deepen the sense of marginalization (Saif et al., 2020). Their minority identity, shaped by exclusion, discrimination, and the complex negotiation of ties to the Arab world, Palestinian identity, and Israeli citizenship, complicates their sense of belonging and access to meaningful civic engagement opportunities (Chaider et al., 2010; Miaari and Hadad Haj-Yahya, 2017).

### Civic engagement as a means for development

Theories of positive youth development (PYD; Balsano, 2005; Lerner et al., 2009), positive psychology (Egan et al., 2008), and sociopolitical anti-oppression and critical pedagogical theories (Ballard and Ozer, 2016) point to civic engagement as a key strategy for enhancing youth development and wellbeing (Jenkinson et al., 2013). In the PYD framework, development results from interactions between individuals and social contexts, with community engagement playing a crucial role in fostering the 5Cs of competence, confidence, connection, caring, and character that lead to the sixth C, contribution (Lerner et al., 2005; Wray-Lake et al., 2019). This view does not only see civic engagement not as a resource but as a developmental context for the 5Cs. The extension to the sixth C of contribution creates a link between civic participation and development that is particularly pertinent to studying civic engagement in socially marginalized young adults.

Building on this developmental perspective, positive psychology further explicates the psychological mechanisms through which civic engagement contributes to individual wellbeing. Studies have shown that prosocial behavior and community involvement can enhance life satisfaction, meaning in life (Birger-Sagiv et al., 2022a, 2022b, 2025), and hope by promoting agency and goal-directed, agentic thinking (Egan et al., 2008; Birger-Sagiv et al., 2022b). Complementing both the developmental and psychological lenses, theories of anti-oppression and critical pedagogy draw attention to the structural and political dimensions of civic engagement. Rooted in Paulo Freire’s critical philosophy (Freire, 1997/2000), pluralistic and feminist theories (Brooks, 2010; Eagly et al., 2012; Kerner, 2017), and equity-centered theory (Green et al., 2021), these approaches conceptualize civic engagement as a means of empowerment that enables young people to advocate for structural change by promoting equity, amplifying marginalized voices, and promoting social justice (Ballard and Ozer, 2016; Refaeli et al., 2019; Birger-Sagiv et al., 2022a, 2022b; Phan and Kloos, 2022).

Research on the developmental benefits of civic engagement during adolescence and emerging adulthood has predominantly examined samples from the U. S and Europe. These studies consistently highlight the positive impact of civic engagement on mental health in the late twenties and early thirties (Ballard et al., 2018; Ward, 2013; Wray-Lake et al., 2019) that leads to greater hope, life satisfaction, meaning in life, overall wellbeing (Jenkinson et al., 2013; Santini et al., 2019; Son and Wilson, 2015; Webb et al., 2017; Yeung et al., 2017), fewer depressive symptoms, and less criminal behavior, and substance use (Thompson et al., 2024). Civic engagement has also been linked to commitment to future civic and political involvement (Beaumont et al., 2006; Nishishiba et al., 2005).

Civic engagement in marginalized minorities often involves activities that directly benefit their communities (Sánchez-Jankowski, 2002; Ginwright et al., 2006). In the European context, empowerment frameworks have conceptualized this engagement through a personal-cultural-structural lens that promotes self-confidence and leadership while encouraging youth to challenge racism, xenophobia, and other forms of oppression (Haji-Kella, 2000; Council of Europe, Congress of Local and Regional Authorities, 2022). Nonetheless, minority populations, and youth in particular, often have limited access to civic opportunities and participate less frequently than the broader population. This pattern has been linked to distrust in government, experiences of discrimination and exclusion, alienation from institutions, limited resources, and persistent feelings of powerlessness and non-belonging (Brady et al., 2020; Fenn et al., 2021; Phan and Kloos, 2022).

Nevertheless, recent empirical work has begun to shed light on the role of civic engagement in the lives of marginalized minority young adults in more diverse contexts. A qualitative study of Asian American immigrant and second-generation college students found that civic engagement was often initiated through siblings, peers, and community networks, although acculturative tensions sometimes constrained involvement; participation was associated with increased self-confidence, leadership skills, a sense of belonging, and academic adjustment (Chan, 2011). Longitudinal data from low-income racial minority youth in the U.S. (93% African-American) showed that civic engagement in adolescence predicted higher educational attainment, greater civic participation, higher life satisfaction, and lower arrest rates in emerging adulthood (Chan et al., 2014). Similarly, qualitative studies of Arab university graduates in Israel who participated in extra-curricular civic programs indicate that civic engagement fostered identity development, political awareness, and emotional relief from academic and social pressures (Goldner and Golan, 2017; Goldner and Midhat Nagamey, 2019).

Together, these studies suggest that adopting an anti-oppression perspective can help minority populations develop both inner and outer awareness, as well as a deeper understanding of how societal power dynamics and institutional barriers shape their marginalized, intersectional identities. Developing the capacity to critically analyze socio-political hierarchies and power relations, while exploring the interaction between broader societal contexts and individual self-identity, can provide socially excluded volunteers with a more nuanced and empowering sense of self (Birger-Sagiv et al., 2022b; Goldner and Golan, 2017; Cadenas and Kiehne, 2021). Despite the growing emphasis on empowerment-oriented civic engagement, additional research is needed to clarify its benefits for the wellbeing of minority young adults (Chan et al., 2014).

Studies examining civic engagement among minority youth have documented systematic variation in the forms, quality, and intensity of engagement across participant characteristics, including age, gender, education, family support, and personality traits (Elwazani and Khorshidifar, 2024; Bussell and Forbes, 2002; Wilson, 2000; Jugert et al., 2013; Carlo et al., 2005). Women and individuals with higher education and income tend to be more socially engaged, whereas older and more socioeconomically disadvantaged activists often report greater personal and community benefits from volunteering (Bussell and Forbes, 2002; Wilson, 2000; Morrow-Howell et al., 2009). Dispositional factors (e.g., generosity, caring, agreeableness, extraversion), social support, sense of belonging, and technological self-efficacy have all been linked to civic involvement, including online engagement (Reed and Selbee, 2000; Bussell and Forbes, 2002; Jugert et al., 2013; Carlo et al., 2005; Deng and Fei, 2023). Overall, these findings suggest that individual characteristics shape both engagement patterns and the benefits derived from civic participation, potentially moderating its developmental outcomes.

### The current study

To help bridge the gap in the literature, the current study examined to what extent participating in civic engagement contributed to an increase in young Arab adults’ civic engagement attitudes, behaviors and wellbeing (as measured in terms of self-reported meaning in life, self-worth, satisfaction in life, and hope) and a decrease in depression symptoms and loneliness before and after 12 months of involvement in a civic engagement program. It also examined whether young adults with different psychological profiles would benefit differently from participating in this program in terms of their attitudes, perceptions, and behaviors regarding civic engagement.

Drawing on the PYD framework’s Five Cs and anti-oppression theories, it was hypothesized that civic engagement would increase agency and social justice awareness in Arab minority youth while countering systemic marginalization to enhance wellbeing outcomes. In particular, we hypothesized that after participating in the civic engagement program for 12 months, participants would exhibit more positive attitudes toward civic engagement, report greater knowledge and skills in civic engagement and advocacy, and engage in a larger number of civic activities in their daily lives, and that they would report higher levels of meaning in life, as well as greater life satisfaction, self-worth, and hope, and lower levels of loneliness and depression. Due to the absence of previous findings on the contribution of civic engagement in participants with different wellbeing and psychopathology profiles, the latter question was exploratory.

### The setting

The data were collected from the “Meidad Enterprise-Social Engagement in Young Arab Adults” project initiated by the Fund for Special Enterprises of the Israel National Insurance Institute, Israel state welfare bureaus, and the Gandyr and Lautmann Foundations. As part of this project, various organizations established 15 groups of socially excluded young Arab adults nationwide. In most cases, the participants were recruited through welfare bureaus that serve young Arab adults experiencing social and structural exclusion, including limited access to higher education or vocational pathways, and marginalization related to minority status and geographic peripherality.

Note that the role of the welfare bureaus was limited to referrals and mediation. Welfare professionals were asked by the program coordinators to recommend young Arab adults who might benefit from participation in a civic-engagement initiative, based on their professional familiarity with the population, but these welfare bureaus were not involved in the recruitment process itself, the delivery of the program, or the research procedures. After the referrals, all contact with potential participants was conducted by the participating organizations and program coordinators.

This project aimed to encourage civic engagement through an innovative approach that scaffolds participants’ orientations during emerging adulthood to promote future civic engagement and wellbeing. The 12-month program consisted of a couple of weekly two-hour group sessions, led by trained coordinators, with 5–15 participants. It covered modules on active citizenship (such as civic engagement and participatory skills), social justice advocacy (including workshops on systemic inequities), and leadership skills (e.g., group problem-solving activities), all designed to address the socio-political challenges faced by Arab youth. The data collection began in August 2022 and ended in August 2023.

## Method

The current study employed a longitudinal pre-post study design with two measurement points. The initial evaluation took place at the start of the program (T1), and the follow-up assessment was conducted during the program’s last month (T2, approximately 12 months later).

### Participants and procedure

The sample consisted of 132 socially excluded Arab youth (*M*_age_ = 20.40, *SD* = 2.04; 57% women), defined as individuals aged 17–27 with limited access to education or employment. Participants were recruited mainly through welfare bureaus based on predefined criteria, including high school dropout status, unemployment, or residence in marginalized communities. Ethical approval was obtained from the Ethics Committee of the Faculty of Social Welfare and Health Sciences at the University of Haifa (#268/23). Participants were recruited during group meetings and invited to take part in the study. Participation was entirely voluntary, and participants were assured of anonymity and informed that they could decline or withdraw at any stage without repercussions. All participants provided written informed consent prior to participation. Before questionnaire administration, the first author introduced the study’s general aims and ethical guidelines and remained available to address any questions. Data were collected using online questionnaires administered via the Qualtrics platform during group meetings. Questionnaire completion took approximately 30 min.

The current sample was drawn from an initial baseline sample of 261 individuals and completed assessments at two time points. Attrition was primarily due to reduced interest, transitions into employment or educational frameworks, and competing time demands. Recruitment constraints precluded the inclusion of a control group. Although this limits causal inference, pre–post field designs are commonly used in community and clinical research where random allocation is not feasible and can provide valuable insights into feasibility, acceptability, and preliminary efficacy (Cook et al., 2002; Martini et al., 2023; Oubiña López and Gómez Baya, 2025). A power analysis conducted using G*Power (Faul et al., 2007) indicated that the final sample size was sufficient to detect small-to-moderate effect sizes (*μ*^2^ = 0.08–0.13) with 80% power (*α* = 0.05).

A battery of self-report questionnaires was administered during the two assessments to assess the participants’ attitudes, skills, and behaviors toward civic engagement, as well as their wellbeing and distress, with adaptations to ensure cultural relevance for Arab youth facing socio-political marginalization and to align with the study’s focus on empowerment and advocacy. The first two researchers translated the measures into Arabic and back-translated them into English. The translations were reviewed and refined through an iterative process until a final version was agreed upon by all involved. All discrepancies were resolved through consensus. To improve data quality and minimize missing or inconsistent responses, adjustments were made to the questionnaires, including shortening their length (Fenton et al., 2001), simplifying the language (Cha et al., 2007), and tailoring the content to the specific context of this study (Azaiza and Ben-Ari, 1998). To make the response scales easier to use, all the questionnaire items were rated on a five-point Likert scale (1—strongly disagree, 5—strongly agree). Parts of the questionnaires were previously used with Arab at-risk young adults (see Birger-Sagiv et al., 2022a). Two participants did not fully complete the questionnaires; missing data affected fewer than 10% of responses and were not imputed to preserve data integrity (Muhammad, 2023).

In terms of demographics, most participants (97%) were single; the rest were married or divorced. Most respondents (92%) lived with their parents. Of the participants, 61% had a part-time or full-time job, and 98% were Muslim. This high proportion of Muslims in the program was expected, given that Arab Christians constitute only 6.9% of the Arab population in Israel and tend, on average, to have higher educational attainment, including higher rates of secondary school completion (86%) and enrollment/degrees in higher education (53%) (Israel Central Bureau of Statistics, 2024). In the sample, 41% defined themselves as traditional (41%), 42% as religious, 7% as secular, and the rest (2%) as ultra-Orthodox. In terms of years of education 12% had up to high school education without a matriculation certificate, 47% had a high school education with a matriculation certificate, 14% were college students or had a college education, and 17% were high school students. No differences were found between participants who remained in the program and those who dropped out in terms of their background or the study variables.

### Measures

#### Civic engagement measures

##### Favorable attitudes toward civic engagement

Positive attitudes toward civic engagement were assessed using five items from the Volunteer Identity Scale (Callero et al., 1987; Cheung et al., 2015), which evaluates individuals’ pro-civic attitudes and the desire and mindset to get involved with others to make positive contributions to society (e.g., “Civic engagement is an essential part of who I am,” “For me, being socially engaged means more than just volunteering”). The internal reliabilities (Cronbach’s alphas) were reported to range from 0.75 to 0.76 (Cheung et al., 2015, 2016). The Cronbach’s alphas in the current study were 0.64 and 0.72 for T1 and T2, respectively.

##### Civic skills

Three items from the interpersonal and problem-solving skills subscale and two items from the leadership subscale from the Civic Attitudes and Skills Questionnaire (CASQ; Moely et al., 2002) were used to assess the extent to which the participants could work collaboratively, communicate, and successfully solve problems (“I can work cooperatively with a group of people”) and the extent to which they could influence their surroundings effectively (“I have the ability to lead a group of people”). The internal reliabilities (Cronbach’s alphas) for both scales in the original study were 0.79. In addition, four items from the Civic Skills subscale the Civic Identity/Civic Engagement inventory (CICE; Bobek et al., 2009) were utilized to assess the extent to which the participants felt they had the skills and ability to be involved in social and democratic activities (e.g., “What is your ability to sign an e-mail or written petition?”). The internal reliabilities (Cronbach’s alphas) in the current study were 0.79 and 0.74 for T1 and T2, respectively.

##### Participatory skills

Participatory skills were assessed using seven items from the Skills subscale of the Knowledge, Attitudes, Skills, and Practices scale (KASP; Bartlett and Schugurensky, 2024). The participants were asked to rate their participatory civic skills, including leadership skills, goal setting, collaborative problem solving, and time management (e.g., “I can help organize others to solve a problem,” “I can resolve conflicts.”). The overall score was the mean of the items on the questionnaire. The internal reliabilities (Cronbach’s alphas) in the current study were 0.85 for T1 and T2.

##### Social/political awareness

Social/political awareness was assessed using six items measuring political and local understanding of political and national/local issues from the Political Awareness sub-scale of the Civic Attitudes and Skills Questionnaire (CASQ; Moely et al., 2002) (e.g., “I understand the issues facing my city/my community.”) The Cronbach’s alpha for the Political Awareness sub-scale ranged from 0.79 to 0.80 (Moely et al., 2002). The Cronbach’s alpha for the LTSJ-B was reported to be 0.70 (Ginns et al., 2015). The internal reliabilities (Cronbach’s alphas) for the sub-scale in the current study were 0.85 and 0.89 for T1 and T2, respectively.

##### Knowledge of rights and advocacy

Three items developed for the current study evaluated the extent to which the participants were familiar with the range of services and rights they are entitled to (e.g., “I am familiar with the range of social services available in my area of residence, such as social workers, National Insurance, Youth Centers”). The internal reliabilities (Cronbach’s alphas) for the scale in the current study were 0.75 and 0.80 for T1 and T2, respectively.

##### Civic behaviors

The participants were asked to indicate whether they had engaged in political, social, volunteer, and/or environmental involvement over the year (yes/no). These items were based on the Civic Engagement in Young Adulthood Index (Zaff et al., 2008) and the Volunteer Stream Monitoring scale by Overdevest et al. (2004). The items examined the extent to which the participants had engaged in a range of pro-social activities, including signing a petition on political or public issues, participating in a demonstration, writing a response on political or social problems to posts/talkbacks/publications on social networks or websites, volunteering in some way outside of the group’s activities, helping a person who was not a member or relative, donating money to an organization or a person who was not a member or relative, reporting environmental hazards to the municipality (such as a leaking faucet), and taking steps to protect the environment (for example, recycling bottles, saving water, cleaning up outside). The total score was the sum of the positive items. The internal reliabilities (Cronbach’s alphas) for the scale in the current study were 0.72 and 0.65 for T1 and T2, respectively.

##### Voting behavior intentions

Intention to vote was assessed on two items: “Please indicate the extent to which you intend to vote in the next local elections,” and “Please indicate the extent to which you intend to vote in the next national elections.” These items were based on Netemeyer and Burton (1990). The internal reliabilities (Cronbach’s alphas) for the scale in the current study were 0.92 and 0.71 for T1 and T2, respectively.

##### Exercise of rights

Exercise of rights was assessed using four items developed for the current study that evaluated the extent to which the participants exercised their rights in various state institutions, such as the Welfare Department, National Insurance Institute, and youth centers (yes/no) (e.g., “During the previous six months, I contacted the Welfare Department for assistance with housing, employment, etc.”; “During the previous six months, I contacted the Youth Center or another entity for assistance with scholarships or guidance for education.”)

#### Wellbeing and psychopathology measures

##### Meaning in life, satisfaction in life, and self-efficacy

Participants’ wellbeing was assessed using the Meaning and Purpose in Life, Satisfaction in Life, and Self-Efficacy sub-scales from the Comprehensive Inventory of Thriving (CIT; Su et al., 2014). The Meaning in Life Scale assesses the respondent’s goal-directedness or perception of the significance or purposefulness of life (three items, e.g., “I know what gives meaning to my life”). The internal reliabilities (Cronbach’s alphas) for the meaning in life subscale in the current study were 0.82 and 0.86 for T1 and T2, respectively. The satisfaction of life sub-scale assesses the extent to which participants feel content, fulfilled, or happy (three items, e.g., “I am satisfied with my life”). The internal reliabilities (Cronbach’s alphas) for the satisfaction in life sub-scale in the current study were 0.77 and 0.80 for T1 and T2, respectively. The self-worth sub-scale assesses the extent to which participants feel a sense of self-worth and value (three items, “What I do in life is valuable and worthwhile.”) The internal reliabilities (Cronbach’s alphas) for the self-worth sub-scale in the current study were 0.79 and 0.93 for T1 and T2, respectively.

##### Hope

The State Hope Scale (Snyder et al., 1996) was used to assess the extent to which the participants believed in their ability to act, generate effective plans, and find ways to achieve their goals (six items, e.g., “I can think of many ways to reach my current goals.”) The internal reliabilities (Cronbach’s alphas) in the current study were 0.85 and 0.89 for T1 and T2, respectively.

##### Loneliness

Loneliness was assessed using three items from the Three-Item Loneliness Scale (Hughes et al., 2004) (“I feel left out,” “I feel isolated from others,” and “I lack companionship”) and one item from the UCLA-8 Loneliness Scale (ULS-8; Hays and DiMatteo, 1987; “There is no one I feel close to”). These items were designed to measure feelings of loneliness and social isolation using a simplified set of responses (Hughes et al., 2004). The scale was previously used among young adults (Prado et al., 2024), adults in disadvantaged neighborhoods (Jamalishahni et al., 2024), and female army veterans (Williamson et al., 2022). The original study’s internal reliability (Cronbach’s alpha) was 0.72. The internal reliabilities (Cronbach’s alphas) in the current study were 0.92 and 0.93 for T1 and T2, respectively.

##### Depression symptoms

Depression symptoms were assessed on the Patient Health Questionnaire (PHQ-2; Kroenke et al., 2003). The participants were asked to indicate the extent to which they had been bothered by any of the following problems over the last 2 weeks: “Having little interest or pleasure in doing things;” “Feeling down, depressed, or hopeless.” The original study reported the construct and criterion validity of the PHQ-2 (Kroenke et al., 2003). The PHQ-2 has proven validity as a depression screening tool, according to a meta-analysis of 21 studies (*N* = 11,175), including 1,529 participants diagnosed with major depressive disorder using a gold-standard assessment. Especially at a cut-off score of ≥2, the measure showed high pooled sensitivity (0.91) and acceptable specificity (0.70) across 17 studies, supporting its usefulness (Manea et al., 2016). The internal reliabilities (Cronbach’s alphas) in the current study were 0.93 and 0.79 for T1 and T2, respectively.

### Data analysis

The differences between the Time 2 and the Time 1 scores were calculated using four repeated measure MANOVAs on the participants’ civic engagement attitudes and perceptions (positive attitudes toward civic engagement, civic skills, participatory skills, social/political awareness, knowledge of rights and advocacy), participants’ civic engagement behaviors (voting behaviors, exercise of rights, civic behaviors), participants’ wellbeing (meaning in life, satisfaction in life, and self-worth), and psychopathology (loneliness and depression). These analyses were followed by an ANOVA with repeated-measures. In addition, we conducted an ANOVA on participants’ hope.

To identify subgroups of participants based on their wellbeing, hope, and psychopathology at T1, the K-means algorithm (Jaeger and Banks, 2023; Reddy and Vinzamuri, 2018) was used to cluster the participants into three subgroups based on their baseline wellbeing and psychopathology, which were selected to reflect theoretical distinctions in PYD (e.g., high, moderate, low functioning). The elbow method and silhouette analysis were used to determine the optimal number of clusters needed to capture psychological variability (Kuswardana et al., 2025).

In K-Means clustering, the number of clusters (k) is determined by calculating the midpoints, referred to as “centroids,” where each data point is assigned to the centroid of the cluster to which it is the closest. The mean of the values in each group represents the centroid. The procedure begins by mapping each data point onto a coordinate plane based on its associated variable values. Once the points are plotted, the centroid is calculated. Then, the distances between each data point and each centroid are determined, and each point is assigned to the nearest centroid, forming a cluster. Then, the centroids are recalculated based on the newly formed clusters, and the distances from each data point to the updated centroids are computed. The data points are reassigned to the nearest centroid, and this process repeats until no further changes in cluster assignments occur (Reddy and Vinzamuri, 2018).

The decision to use a K-means analysis was dictated by the algorithm’s well-established efficiency in data classification that generates homogeneous clusters within a dataset (Jaeger and Banks, 2023). Psychological constructs such as depression, loneliness, life satisfaction, and meaning in life are often measured on continuous scales, and K-means clustering, which assumes continuous input variables and uses a distance-based metric such as the Euclidean distance to group individuals with similar profiles, is appropriate for this type of data (Steinley, 2006). K-means also enables flexibility in specifying the number of clusters, which researchers can define based on theoretical or practical considerations, such as hypothesizing three distinct subgroups of individuals based on their psychological characteristics. K-means clustering is frequently used in psychological research to classify individuals by psychological traits and outcomes, including grouping individuals with similar levels of wellbeing or mental health profiles (e.g., Liu and Wang, 2021; Liu et al., 2022).

After this cluster analysis, two MANOVA analyses were calculated on civic engagement attitudes and perceptions (political awareness, volunteering identity, civic skills, participatory skills, and knowledge of rights and advocacy) and civic behaviors (civic engagement activities, voting intentions, and exercise of rights) to identify an interaction effect for clusters x measurement time.

## Results

### Changes in participants’ civic engagement

The MANOVA analysis on participants’ civic engagement attitudes and perceptions (positive attitudes toward civic engagement, civic skills, participatory skills, social/political awareness, knowledge of rights, and advocacy) revealed a significant main effect for time of measurement on participants’ civic engagement attitudes and perceptions, *F*(5, 121) = 3.70, *p* ≤ 0.01, *μ*^2^ = 0.13, civic engagement behaviors *F*(3, 125) = 3.96, *p* ≤ 0.01, *μ*^2^ = 0.09, As indicated in Table 1, the ANOVA for civic engagement attitudes and perceptions showed a significant increase in participants’ social/political awareness, civic skills, and knowledge of rights and advocacy. The ANOVA for civic engagement behaviors indicated a significant increase in participants’ exercise of rights and a marginally significant increase in civic engagement activities and behaviors.

**Table 1:** Results of the ANOVA analyses.

Variable	*M*T1	*M*T2 *SD*	*d*’	*F*
	*SD*		*Cohen*	*μ* ^2^
	*Range*			
Social/political awareness	3.77	4.02	0.35	10.29**
	0.70	0.72		0.08
	1.00–5.00	1.00–5.00		
Positive attitudes and perceptions toward civic engagement	3.72	3.81	0.13	1.94
	0.70	0.70		0.02
	1.00–5.00	1.00–5.00		
Civic skills	3.41	3.65	0.28	8.69**
	0.85	0.85		0.07
	1.00–5.00	1.00–5.00		
Participatory skills	3.99	3.99	0.00	0.02
	0.61	0.55		0.00
	1.00–5.00	1.00–5.00		
Knowledge of rights and advocacy	3.41	3.69	0.37	9.63**
	0.85	0.67		0.07
	1.00–5.00	1.00–5.00		
Civic behaviors	3.36	3.77	0.21	3.62#
	2.07	1.87		0.03
	0.00–8.00	0.00–8.00		
Voting intentions	1.53	1.65 0.70	0.16	1.94
	0.8	0.00–2.00		0.02
	0.00–2.00			
Exercise of rights	2.12	2.47	0.32	9.29**
	1.09	1.13		0.07
	1.00–5.00	1.00–5.00		
Meaning in life	3.93	4.17	0.33	10.04**
	0.77	0.67		0.07
	1.33–5.00	2.00–5.00		
Satisfaction in life	3.61	3.81	0.25	6.11*
	0.83	0.76		0.05
	1.00–5.00	2.33–5.00		
Self-worth	3.93	4.02	0.12	1.33
	0.8	0.7		0.01
	1.00–5.00	1.00–5.00		
Loneliness	2.15	1.98	−0.18	3.58#
	0.96	0.95		0.03
	1.00–5.00	1.00–5.00		
Depression symptoms	2.08	2.04	−0.04	0.25
	0.98	0.95		0.00
	1.00–5.00	1.00–5.00		
Hope	3.83	3.91	0.13	1.62
	0.71	0.56		0.01
	1.00–5.00	2.83–5.00		

^#^*p* ≤ 0.10, *p ≤ 0.05, **p ≤ 0.01.

### Changes in participants’ wellbeing and psychopathology

The MANOVA analysis on participants’ wellbeing revealed a significant main effect for time of measurement on participants’ wellbeing *F*(3, 125) = 3.70, *p* ≤ 0.05, *μ*^2^ = 0.08. By contrast, the MANOVA for psychopathology and the ANOVA for participants’ hope did not indicate significant changes from baseline. The ANOVA for the participants’ wellbeing indicated significant increases in meaning in life and life satisfaction. In contrast, the ANOVA for participants’ psychopathology indicated a marginally significant decrease in participants’ loneliness (see Table 1).

### Participants’ development based on their level of wellbeing, Hope, and psychopathology

The K-means cluster analysis yielded three distinct groups (see Table 2). This provided a more granular picture of the aggregation of participants’ psychological characteristics, with a reasonable number of participants in each cluster. The first cluster was characterized by the highest level of functioning, meaning the lowest level of psychopathology and the highest level of wellbeing and hope (*n* = 51, 61% women, *M*_age_ = 20.35), and was labeled “well-adjusted, thriving.” The second cluster showed the highest level of psychopathology, along with moderate wellbeing and hope, and was labeled “Distressed but Resourceful” (*n* = 28, 57% women, *M*_age_ = 20.13). The third cluster had a moderate level of psychopathology and the lowest levels of wellbeing and hope, and was labeled “Low Distress but Low Wellbeing” (*n* = 51, 63% women, *M*_age_ = 20.79).

**Table 2:** Results of cluster analysis according to participants’ psychopathology and wellbeing.

Group	1 Well-Adjusted, Thriving (*n* = 51)	2 Distressed but Resourceful (*n* = 28)	3 Low Distress but Low Wellbeing (*n* = 51)
Variable Loneliness	1.36	3.29	2.29
Depression	1.41	3.21	2.14
Self-worth	4.47	4.24	3.25
Meaning in life	4.49	3.87	3.42
Satisfaction with life	4.17	3.67	3.05
Hope	4.28	3.93	3.32

As shown in Table 3, which details cluster-specific improvements, although the MANOVA on civic engagement attitudes and perceptions revealed main effects for measurement time, *F*(5, 120) = 3.02, *p* ≤ 0.05, *μ*^2^ = 0.12, and for groups, *F*(5, 120) = 5.31, *p* ≤ 0.001, μ^2^ = 0.18, with participants in Clusters 1 and 2 scoring higher than Cluster 3 at both T1 and T2, the significant group × time interaction, *F*(5, 120) = 6.47, *p* ≤ 0.001, *μ*^2^ = 0.21, showed that Cluster 3 achieved the largest gains in civic skills, participatory skills, and self-advocacy knowledge (see Table 3).

**Table 3:** ANOVA analyses according to participants’ groups.

	Group 1		Group 2		Group 3		*F* time *μ*^2^	*F* groups *μ*^2^	*F* interaction *μ*^2^	Contrasts
	High Functioning (*n* = 51)		High in psychopathology and Med in wellbeing		Med in psychopathology and low in wellbeing					
Variable	*M*T1	*M*T2	*M*T1	*M*T2	*M*T1	*M*T2				
	*SD*	*SD*	*SD*	*SD*	*SD*	*SD*				
Political awareness	3.88	3.77	3.87	3.96	3.6	4.04	9.30**	0.51	2.5	
	0.61	0.71	0.76	0.8	0.74	0.73	0.07	0	0.04	
Positive attitudes	4	4	3.59	3.77	3.52	3.65	2.25	6.77**	0.66	
	0.65	0.78	0.53	0.65	0.76	0.61	0.02	0.1	0.01	
Civic skills	3.72	3.72	3.52	3.69	3.05	3.56	0.02	6.51**	3.87*	1 > 3
	0.68	0.59	0.79	0.67	0.9	0.69	0	0.1	0.06	
Participatory skills	4.24	4.05		4.13	3.66	3.85	8.01**	9.95***	5.18**	1 > 2,3
	0.53	0.58	4.11	0.57	0.55	0.49	0.06	0.14	0.08	
			0.61							
Knowledge of rights and advocacy	3.65	3.66	3.78	3.71	2.95	3.7	7.61**	6.44**	10.96***	1 > 2,3
	0.72	0.62	0.71	0.7	0.86	0.71	0.06	0.1	0.15	
Civic behaviors	3.82	3.75	3.74	3.78	2.69	3.79	2.67	1.75	3.39*	
	1.86	1.59	2.19	2.19	2.05	2	0.02	0.03	0.05	
Voting intentions	1.45	1.71	1.7	1.56	1.52	1.62	0.77	0.08	1.85	
	0.84	0.67	0.67	0.8	0.82	0.67	0.01	0	0.03	
Exercise of rights	2.18	2.82	2.56	2.66	1.82	2.46	7.08**	2.33*	2.09	2 > 3
	1.23	1	1.17	1.35	0.78	1.02	0.06	0.04	0.03	

*p ≤ 0.05, **p ≤ 0.01, ***p ≤ 0.001.

Likewise, the MANOVA for civic behaviors (civic engagement behaviors, voting intentions, exercise of rights) revealed an interaction effect for clusters x measurement time, *F*(3, 120) = 3.08, *p* ≤ 0.05, *μ*^2^ = 0.07. The *t-test* analyses showed that participants in Cluster 3 evidenced the largest gains in the number of civic behaviors throughout the year, *t*(48) = −5.01, *p* ≤ 0.000, 95%CI [−0.90, −0.38] (see Table 3).

## Discussion

This study examined the impact of civic engagement programs on socially excluded and at-risk Indigenous Arab young adults in Israel over a 12-month period. The study was designed to examine changes in participants’ civic engagement attitudes and behaviors, while also assessing the program’s impact on overall wellbeing, including meaning in life, self-worth, life satisfaction, and hope. It analyzed how individual psychological factors shaped the development of civic attitudes and participatory behaviors throughout the program.

The findings are consistent with cross-sectional surveys (Birger-Sagiv et al., 2022b; Santini et al., 2019; Yeung et al., 2017) and qualitative studies on the crucial role of civic engagement in young adults’ wellbeing and thriving (Birger-Sagiv et al., 2025; Webb et al., 2017), as well as qualitative (Goldner and Golan, 2017; Sze-Yeung Lai and Chi-leung Hui, 2021) and longitudinal studies (Beaumont et al., 2006; Nishishiba et al., 2005) that have demonstrated the development of prosocial behavior, sustained civic participation, and political engagement.

Lerner et al. (2005) defined positive youth development (PYD) as encompassing five key attributes, referred to as the “Five Cs”: The first attribute, Competence, includes social, academic, and vocational skills. The second is Confidence, which reflects a positive sense of self; the third is a positive connection with family, peers, and the community. The fourth attribute, Character, pertains to moral values, integrity, and the principles that promote personal and communal health; lastly, compassion or caring highlights empathy toward others. After possessing all the first five attributes, a “sixth C” Contribution arises (Little, 1993; Pittman et al., 2001); as youth develop the first five Cs, they are more willing to contribute to their community (Lerner et al., 2003). However, the development of the five Cs does not occur in isolation, and the available resources and support influence it, ultimately shaping the healthy trajectory of youth (Leffert et al., 1998). Consistent with this framework, our initial findings indicated positive outcomes across several areas: enhanced civic engagement skills and advocacy knowledge (Competence), prosocial behaviors (Character), and increased meaning in life and life satisfaction (Confidence).

The psychological benefits of civic engagement were analyzed here through the framework of Positive Youth Development. However, the findings can also be understood through broader theories of prosocial behavior. Research has found that acts of kindness generate positive emotional experiences (Fredrickson, 2013; Lyubomirsky and Layous, 2013; Scarf et al., 2016) by producing what Andreoni (1989) termed the “warm glow” effect, a pleasant emotional reward accompanying prosocial behavior. Individuals who devote time and energy to helping others experience these hedonic feelings as personal rewards (Dunn and Schweitzer, 2005) that can evolve into more profound eudaimonic wellbeing, such as positive emotions that expand cognitive horizons and foster psychological strengths, including personal growth and resilience (Fredrickson, 2013; Sheldon, 2018; Sheldon and Lyubomirsky, 2006).

However, marginalized minorities face significant disparities in access to and participation in civic engagement (Fenn et al., 2021; Webb et al., 2017) that stem from systemic barriers, including institutional distrust, social exclusion, and pervasive powerlessness (Phan and Kloos, 2022). This pattern is particularly evident among young Arabs in Israel, who encounter discrimination, political alienation, exclusion, animosity, and scant access to resources, which collectively impede their community participation (Goldner and Midhat Nagamey, 2019; Goldner and Golan, 2017). The findings suggest that engaging in structured, organized civic activities enables young Arabs to express their voices more effectively, assert their rights, and navigate their communities.

Although participants showed gains in many areas, their self-related variables (self-worth and hope) and pathological indices remained unchanged (*F* not significant). This warrants careful consideration in terms of program design and effectiveness and may reflect the program’s focus on civic skills over mental health interventions, compounded by Arab youth’s exposure to chronic discrimination, suggesting a need for integrated psychological support.

More specifically, self-worth refers to positive or negative self-evaluations encompassing perceptions of successes and failures, as well as emotional regulation (Alarcon et al., 2013). It develops through repeated experiences at both micro and macro levels, and is resistant to change (De Ruiter et al., 2017). For individuals from less privileged backgrounds, discrimination often leads to heightened psychological stress and diminished self-esteem (Aneshensel, 2009; Meyer, 2003; Harris-Britt et al., 2007; Seaton et al., 2010; Umaña-Taylor and Updegraff, 2007). Ongoing exposure to microaggressions and racism may therefore account for the lack of improvement in self-worth and pathology measures observed here, since healthy self-esteem is known to buffer psychological distress and hopelessness (Chioqueta and Stiles, 2007).

Hope also showed resistance to change in the current study, consistent with studies indicating that hope is a remarkably stable construct that develops early in life and remains relatively constant throughout childhood and adolescence (Goldner et al., 2015; Snyder, 1994, 2005). Conceptualized as a form of “control of destiny” (Syme, 2004), hope reflects an active sense of agency that enables individuals to navigate daily stressors. For marginalized youth, this sense of agency is often compromised. Structural inequities, compounded by persistent exposure to negative stressors and limited coping resources, may have constrained the participants’ perceived capacity to influence their lives and futures (Duncan-Andrade, 2009).

The findings also revealed a multifaceted relationship between individual wellbeing and outcomes of civic engagement. Participants with higher initial wellbeing evidenced more sustained civic engagement throughout the year, suggesting that emotional stability, a sense of purpose, and strong social connections facilitate meaningful community involvement. More generally, these findings may point to a bidirectional relationship between mental health and the benefits of civic engagement. Ding et al. (2015) found that participants with poor prior mental health derived fewer benefits from civic participation than those who were initially healthier, most likely because mental health challenges create obstacles across multiple life domains, in particular in forming and maintaining relationships.

At the same time, the program was particularly beneficial for individuals who, although not experiencing severe psychopathology, reported low self-worth, limited fulfillment, diminished meaning, and reduced life satisfaction. This pattern aligns with broader research on interventions. DuBois et al.’s (2011) meta-analysis of mentoring programs revealed a curvilinear relationship between risk and outcomes, suggesting optimal benefits occur at moderate risk levels. Callina et al. (2014) found that moderate hope and varying levels of trust predicted the highest civic engagement among adolescents.

### Practical implications

The findings suggest that participation in civic activities by minority youth living in areas of ongoing political conflict is associated with greater flourishing and more sustained civic engagement over time, thereby highlighting the potential of such programs to promote personal development and broader social involvement. This implies that program designers and leaders should provide both initial and ongoing training that is sensitive to the sociopolitical realities of minority groups and incorporates elements of social justice. Addressing systemic deprivation and power dynamics can help young adult minorities align their personal goals with broader societal issues, fostering a sense of purpose and greater wellbeing. Policymakers, healthcare professionals, and social workers should allocate resources to establish structured civic engagement incubator programs that promote social inclusion, wellbeing, and hope among disadvantaged minority youth. Evidence indicates that volunteering between 40 and 100 h annually provides the greatest health benefits, with no additional gains beyond this range (Musick et al., 1999; Luoh and Herzog, 2002). Accordingly, the findings could inform Israel’s Ministry of Education and Welfare policies, including the National Program for At-Risk Youth, by integrating civic engagement modules into existing frameworks.

At the same time, the findings pointed to issues that may prevent participants from fully benefiting from civic activities, particularly the assumption that these at-risk young adults enter the program with sufficient psychological resources. The lack of amelioration in self-worth and hope suggests that civic engagement alone may not be sufficient to shift deeply rooted self-related constructs, which are shaped by long-term exposure to discrimination and racism. This underscores the importance of incorporating targeted elements into programs that specifically address self-esteem, agency, and hope. The participants who benefited most were those with relatively low self-reported levels of depression and loneliness but diminished self-related resources, indicating that civic engagement may be especially effective for individuals with psychological “room to grow.” For these participants, engagement can act as a catalyst for identity development, meaning-making, and increased self-efficacy, whereas those experiencing significant distress may require additional therapeutic support to participate fully and benefit. Overall, these results emphasize the importance of tailoring civic engagement programs to different psychological profiles and integrating mental health support alongside civic opportunities.

### Limitations and future directions

Several limitations of this study should be acknowledged. First, the study employed a pre-posttest design without a control group, thus limiting causal inference. Second, the effect sizes of the observed correlations were relatively small according to Cohen’s (1988) conventions, suggesting that other participant characteristics, such as attachment security, personality, cognitive abilities, life circumstances, and maturation, may have mediated or moderated the changes. Future research should therefore incorporate control groups and examine these characteristics when predicting development. Third, the sample size was relatively small, reflecting the hurdles involved in recruiting at-risk Arab participants and the substantial attrition rate. These factors warrant caution regarding potential selection bias, limit the generalizability of the findings, and highlight the need for replication with larger samples and a lower dropout rate. Fourth, all measures relied on self-report questionnaires, without additional informants or methods. This raises concerns about social desirability bias, particularly among Arab youth sensitive to stigmatization. Future studies should employ multi-informant designs (e.g., practitioner or facilitator reports) and complementary methodologies such as interviews and observations to enhance validity. Fifth, since baseline levels of depression and loneliness were already low, a floor effect (Liu and Wang, 2021) may have masked the intervention’s potential impact. Future studies should use more sensitive measures or target participants with higher baseline symptoms to detect changes. Finally, to better capture the participants’ development, this study would have benefited from the inclusion of qualitative results, which could have provided more insights into the numerical data, and a more intuitive lay understanding of the findings.

Nevertheless, despite these limitations, the findings indicate that involvement in civic engagement programs can promote greater wellbeing and potentially foster future civic engagement among young minority adults, thereby contributing to the knowledge base on populations experiencing political and social conflict, who are currently underrepresented in the literature.

## Data Availability

The raw data supporting the conclusions of this article will be made available by the authors without undue reservation.
